# The Combination of Natural Compounds Escin–Bromelain–Ginkgo Biloba–Sage Miltiorrhiza (EBGS) Reduces Platelet Adhesion to TNFα-Activated Vascular Endothelium through FAK Signaling

**DOI:** 10.3390/ijms25179252

**Published:** 2024-08-26

**Authors:** Maria Magdalena Barreca, Stefania Raimondo, Alice Conigliaro, Sergio Siragusa, Mariasanta Napolitano, Riccardo Alessandro, Chiara Corrado

**Affiliations:** 1 Department of Biomedicine, Neuroscience and Advanced Diagnostics (Bi.N.D.), Biology and Genetics Section, University of Palermo, 90133 Palermo, Italy; mariamagdalena.barreca@unipa.it (M.M.B.); stefania.raimondo@unipa.it (S.R.); alice.conigliaro@unipa.it (A.C.); riccardo.alessandro@unipa.it (R.A.); 2 Department of Health Promotion, Mother and Child Care, Internal Medicine and Medical Specialties, Haematology Section, University of Palermo, 90127 Palermo, Italy; sergio.siragusa@unipa.it (S.S.); mariasanta.napolitano@unipa.it (M.N.)

**Keywords:** escin, bromelain, ginkgo biloba, sage miltiorrhiza, EBGS, tumor necrosis factor α, endothelial cells, platelet adhesion

## Abstract

Thrombosis is a key process that determines acute coronary syndrome and ischemic stroke and is the leading cause of morbidity and mortality in the world, together with cancer. Platelet adhesion and subsequent activation and aggregation are critical processes that cause thrombus formation after endothelial damage. To date, high hopes are associated with compounds of natural origin, which show anticoagulant action without undesirable effects and can be proposed as supportive therapies. We investigated the effect of the new combination of four natural compounds, escin–bromelain–ginkgo biloba–sage miltiorrhiza (EBGS), on the initial process of the coagulation cascade, which is the adhesion of platelets to activated vascular endothelium. Our results demonstrated that EBGS pretreatment of endothelial cells reduces platelet adhesion even in the presence of the monocyte–lymphocyte population. Our data indicate that EBGS exerts its effects by inhibiting the transcription of adhesion molecules, including P-selectin, platelet membrane glycoprotein GP1b, integrins αV and β3, and reducing the secretion of the pro-inflammatory cytokines interleukin 6, interleukin 8, and the metalloproteinases MMP-2 and MMP-9. Furthermore, we demonstrated that EBGS inhibited the expression of focal adhesion kinase (FAK), strictly involved in platelet adhesion, and whose activity is correlated with that of integrin β3. The results shown in this manuscript suggest a possible inhibitory role of the new combination EBGS in the reduction in platelet adhesion to activated endothelium, thus possibly preventing coagulation cascade initiation.

## 1. Introduction

Cardiovascular disease (CVD), such as atherosclerosis, is the most prominent cause of morbidity and mortality across the world [1]. An important player in acute myocardial events and atherosclerotic progression is thrombosis which occurs in the case of atherosclerotic plaque rupture and activation of the clotting cascade [2,3]. During the thrombosis, the blood clot can completely obstruct the flow through the artery, and thus, many pharmacotherapies combating coronary artery disease, such as anticoagulants and anti-platelets, are targeted toward this process [4]. It is known that inflammation is one of the main pathophysiological mechanisms driving thrombosis initiation and atherosclerosis progression. To date, several studies reported that tumor necrosis factor-alpha (TNFα), a pro-inflammatory cytokine of the TNF/TNFR cytokine superfamily, is physiologically released by tissue macrophages, finally inducing a coagulation cascade that activates a pro-thrombotic state of endothelial cells [5,6,7,8,9,10]. 

Endothelial cells (ECs) form a monolayer, which lines the blood vessels. These cells are involved in maintaining blood fluidity and providing controlled vascular hemostasis at sites of injury. Under physiological conditions, vascular endothelial cells impede platelet adhesion; moreover, ECs constitute a non-adhesive surface that prevents platelet activation and the coagulation cascade. Circulating platelets remain dormant and inactivated thanks to the physiologic anticoagulant and antithrombotic activity of ECs. At sites of vascular damage, ECs acquire a procoagulant phenotype, and the vasoconstrictive function takes over the vasodilatory ones [11]. 

The site of atherosclerotic lesions is characterized by the expression of several extracellular matrix proteins and the recruitment of circulating platelets to the vascular wall. Importantly, activated platelets can improve the local thrombus formation by secreting matrix metalloproteinases (MMPs), such as MMP-2 and MMP-9, thus inducing extracellular matrix degradation and thrombus formation [12,13].

Platelets immediately react by adhering to the exposed extracellular matrix; this first event is followed by platelet–platelet interaction to form a clot that effectively seals the wall of the damaged vessel. In this process, endothelial cells activate a pro-inflammatory phenotype and function as a support for the formation of procoagulant complexes. After platelet adhesion to the vessel wall, the platelets become activated by changing their shape and releasing a plethora of chemokines into the blood or local environment [14]. The initial interaction is mainly mediated by P-selectin or platelet membrane glycoprotein (GP) 1b-IX-V complex to endothelial factors [15]. Furthermore, integrins such as αIIβ3 and αVβ3 are responsible for the stronger and more stable adhesion of platelets [16,17]. Subsequently, platelets change their shape; filopodia and lamellipodia appear on their surface, and finally, platelets increase their adhesion to the injured endothelium, thus preventing other blood loss. Also, the cellular microenvironment constantly affects endothelial cells’ morphology. For example, the regulation of endothelial cell shape is crucial for the correct platelet adhesion, and several compounds may affect this cell shape, inducing cell protrusions, lamellipodia, filopodia, or microvilli-like structure in the endothelium [18]. 

Moreover, platelet–endothelium interaction is influenced by the presence of soluble factors released not only by the activated endothelium but also by monocytes [19,20]. 

Science is making important advances, and we are coming closer to personalized and precision medicine; however, the major problem remains the high toxicity of several drugs, especially toward healthy cells. For this reason, modern pharmacology tries to find new biologically active compounds with lower collateral effects. Growing evidence suggests the clinical possible use of natural compounds as an alternative or combinational therapy with conventional drugs in fields such as cancer, neurodegeneration, inflammation, and cardiovascular diseases [21,22,23,24].

Escin (E) is a mixture of acylated triterpene glycosides isolated from the seeds of horse chestnut (*Aesculus hippocastanum* L.). Varinska and collaborators demonstrated the antiangiogenic properties of escin on HUVEC cells as well as in vivo models [25]. The anti-inflammatory effects of escin and its protective effects on vessel permeability have been known for many years [26,27,28]. Bromelain (B) is the major sulfhydryl proteolytic enzyme extracted from the stem of *Ananas comosus* L. [29]. Several manuscripts describe its anti-inflammatory and cardioprotective activity, and for this reason, bromelain is nowadays introduced into the diet as a supplement [29,30,31]. Ginkgo biloba (G), extracted from the leaves of this plant, and sage miltiorrhiza (S), extracted from its root, have been commonly used in traditional Chinese medicine since ancient times due to their several beneficial effects, such as promoting capillary blood circulation and being cardioprotective, hepato-protective, antiangiogenic, and anti-inflammatory [32,33,34,35].

In this study, we investigate the effects of a new combination of the four natural compounds described above, escin–bromelain–ginkgo biloba–sage miltiorrhiza, and we call it EBGS, as described in the MM section. In particular, we evaluated the effects of EBGS on platelet adhesion to TNFα-activated vascular endothelium

## 2. Results

### 2.1. The Pretreatment of HUVEC with Escin, Bromelain, Ginkgo Biloba, or Sage Miltiorrhiza Does Not Affect the Cell Viability of Endothelial Cells

**Figure 1:**
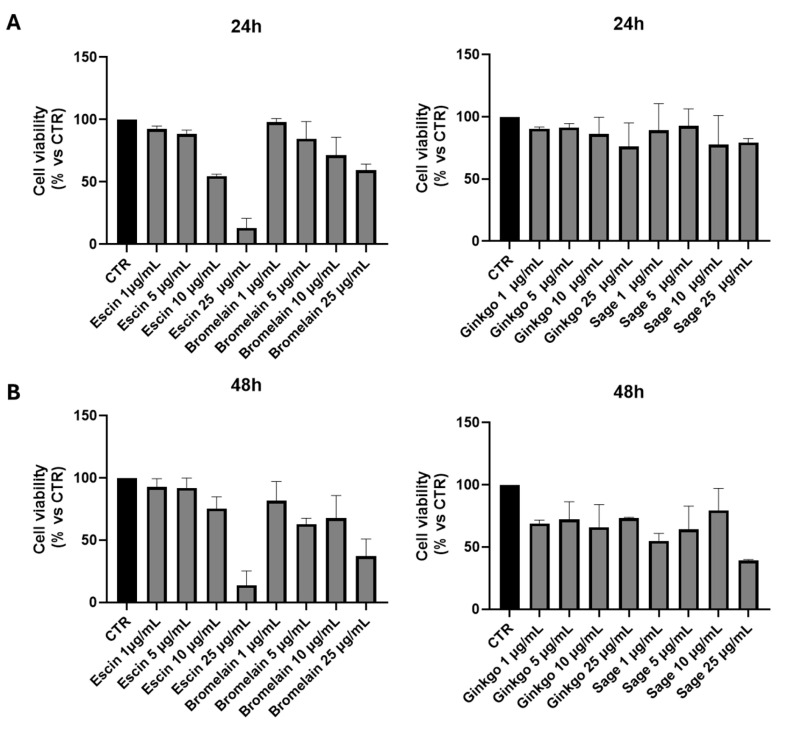
Cell viability assay of HUVEC treated or not for 24 h (**A**) and 48 h (**B**) with increasing concentration (1–5–10–25 µg/mL) of escin, bromelain, ginkgo biloba (Ginkgo), and sage miltiorrhiza (sage). CTR, cells not treated.

To investigate the subtoxic dose of each single compound on HUVEC cells, we performed a viability assay. For this purpose, HUVEC endothelial cells have been treated overnight with escin up to 50 μg/mL, bromelain, ginkgo biloba, and sage miltiorrhiza up to 25 μg/mL. Figure 1 shows that all four compounds start to reduce cellular viability, probably due to low toxicity, even at the dose of 1 μg/mL at 24 h of treatment. These effects seem to increase for all single treatments at 48 h.

For this reason, we decided to perform another cell viability assay treating HUVEC with 0.25 μg/mL of each compound and their combination to have in the combination a maximum concentration of 1 μg/mL. The combination, called EBGS, is the mix of each of the four extracts in equal amounts (0.25 μg/mL of escin, 0.25 μg/mL of bromelain, 0.25 μg/mL of ginkgo biloba, 0.25 μg/mL of sage miltiorrhiza).

**Figure 2:**
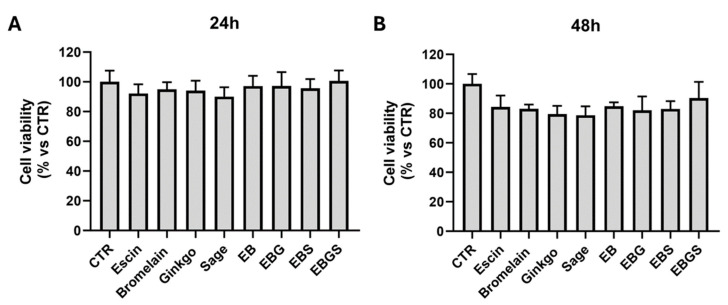
Cell viability assay of HUVEC treated or not for 24 h (**A**) and 48 h (**B**) with 1 μg/mL of each compound or with different combinations as specified: EB, 0.25 μg/mL of escin plus 0.25 μg/mL of bromelain; EBG, 0.25 μg/mL of escin plus 0.25 μg/mL of bromelain plus 0.25 μg/mL of ginkgo biloba; EBS, 0.25 μg/mL of escin plus 0.25 μg/mL of bromelain plus 0.25 μg/mL of sage miltiorrhiza; EBGS, 0.25 μg/mL of escin plus 0.25 μg/mL of bromelain plus 0.25 μg/mL of ginkgo biloba plus 0.25 μg/mL of sage miltiorrhiza. CTR, cells not treated.

In Figure 2, we show that the single treatment and their combination EBGS are well-tolerated by HUVECs. We obtained subtoxic doses of each compound and their combination EBGS. 

Our interest, as explained before, is to evaluate the effects of the new combination EBGS on platelet adhesion to TNFα-activated vascular endothelium. For this reason and due to the results shown, we decided to perform the subsequent experiments with the EBGS combination at the concentration described. Moreover, the subsequent experiments will be conducted for a period of 18 h to maintain the subtoxic effect of the mixture EBGS.

### 2.2. The Pretreatment of HUVEC with EBGS Decreases Platelet Adhesion on Activated Endothelium

We performed the experiment described to demonstrate the potential inhibitory effect of EBGS on the activation of the coagulation cascade. For this purpose, we pretreated HUVEC with each single compound and the EBGS combination, and we activated the endothelium with an inflammatory stimulus (20 ng/mL of TNFα for 2 h). After that, we performed an adhesion assay by seeding the patient’s platelets on the endothelial monolayer.

**Figure 3:**
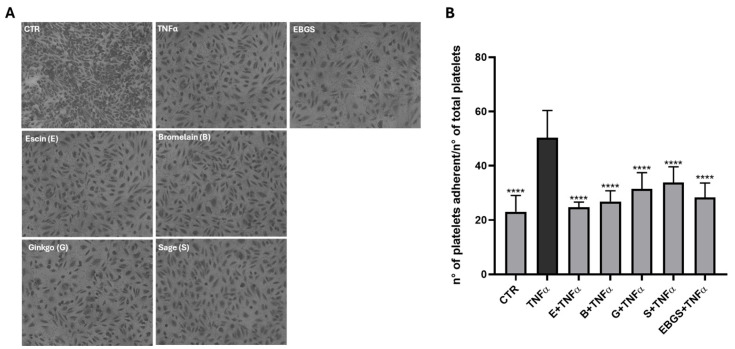
Adhesion assay of patient’s platelets on HUVEC monolayer pretreated or not with single compounds or with EBGS combination and activated by TNFα. (**A**) Representative images of adhesion assay. (**B**) Quantitative analysis of platelets adherent to endothelial monolayer expressed as number of platelets adhered to endothelial cells/number of total platelets for each field. Escin (E), bromelain (B), ginkgo biloba (G), sage miltiorrhiza (S). EBGS: escin–bromelain–ginkgo biloba–sage miltiorrhiza. CTR, cells not treated. Statistical analyses were performed using normality test and *t*-test; **** *p* < 0.0001.

Results showed in Figure 3 confirm that TNFα treatment activates endothelium, thus increasing platelet adhesion; interestingly, pretreatment of EBGS reduces the platelet adhesion on the activated monolayer, suggesting a possible inhibitory role of EBGS in the coagulation cascade.

### 2.3. The Pretreatment of HUVEC with EBGS Reduces the Expression of the Adhesion Molecules Involved in the Platelet Adhesion on Activated Endothelium

**Figure 4:**
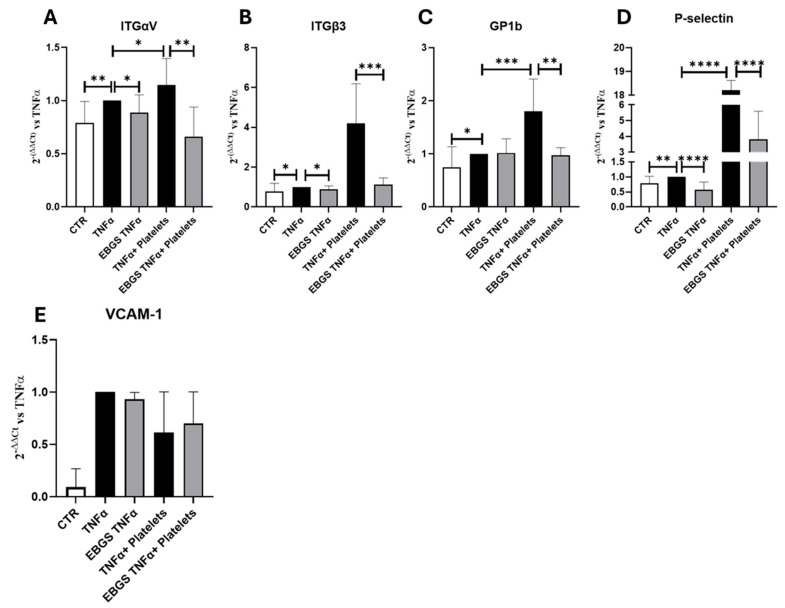
Real-time PCR for integrin αV (ITG αV) (**A**), integrin β3 (ITGβ3) (**B**), platelet membrane glycoprotein GP1b (**C**), P-selectin (**D**), and VCAM-1 (**E**) in HUVEC pretreated or not with EBGS, activated with TNFα, and finally subjected to platelet adhesion. Data were normalized for β-actin. ΔΔCt is expressed as a fold of increase (FOI) to the TNFα-treated sample. Data are expressed as the mean ± SD of at least three independent experiments. EBGS: escin–bromelain–ginkgo biloba–sage miltiorrhiza. CTR, cells not treated. Statistical analyses were performed using normality test and *t*-test; * *p* < 0.05; ** *p* < 0.005; *** *p* < 0.0005; **** *p* < 0.0001.

We intend to investigate the role of EBGS pretreatment on the expression of P-selectin and platelet membrane glycoprotein GP1b, adhesion molecules involved in the initial platelet–endothelium interaction, and on integrins αV and β3, involved in stronger and stable adhesion of platelets. Transcriptional assays demonstrated that TNFα treatment, through endothelium activation, increases the expression of these adhesion molecules. The pretreatment of cells with EBGS reduces the mRNA levels of these molecules (Figure 4). Furthermore, we performed a real-time PCR for the expression of vascular cell adhesion molecules 1 (VCAM-1); results show that VCAM-1 increases with TNFα treatment, but it is not modulated by EBGS even in the presence of platelets.

### 2.4. The Pretreatment of HUVEC with EBGS Reduces the Expression of Inflammatory Chemokines and Metalloproteinases

Interleukin 6 (IL6) and interleukin 8 (IL8) are crucial mediators of inflammation.

**Figure 5:**
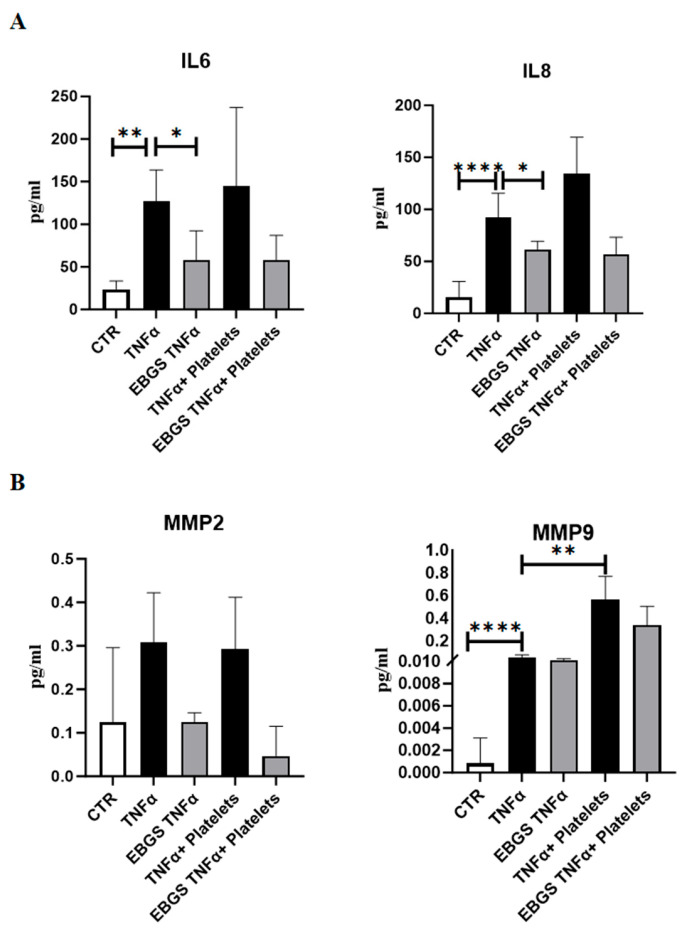
ELISA assay for secreted proteins was performed with conditioned media of HUVEC pretreated or not with EBGS, activated with TNFα, and finally subjected to platelet adhesion. (**A**) ELISA assay for secreted IL6 (left) and for secreted IL8 (right). (**B**) ELISA assay for secreted MMP-2 (left) and for secreted MMP-9 (right). Data expressed as protein concentration (pg/mL) are the mean ± SD of at least three independent experiments. EBGS: escin–bromelain–ginkgo biloba–sage miltiorrhiza. Statistical analyses were performed using normality test and *t*-test; * *p* < 0.05; ** *p* < 0.005, **** *p* < 0.0001.

As expected, the Elisa assays shown in Figure 5A confirmed that TNFα treatment, alone or in combination with platelets, promoted the release of these two inflammatory cytokines in endothelial cells. Interestingly, EBGS pretreatments impede the activation of the inflammatory cytokines mediated by TNFα; the same trend can be observed in the presence of platelets.

To investigate the possible involvement of matrix metalloproteinases in the platelet adhesion to pretreated endothelium, we evaluated MMP expression through the Elisa assay. Interestingly, EBGS pretreatment on activated endothelium reduces the amount of MMP-2 secreted, both with and without platelets. Moreover, platelet recruitment can significantly increase the expression of MMP-9 on activated endothelium (Figure 5B). 

### 2.5. The Pretreatment of HUVEC with EBGS Reduces the Expression of the Adhesion Molecules Focal Adhesion Kinase (FAK) and Vascular Cell Adhesion Molecule 1 (VCAM-1), Involved in Platelet–Endothelial Cell Interaction

**Figure 6:**
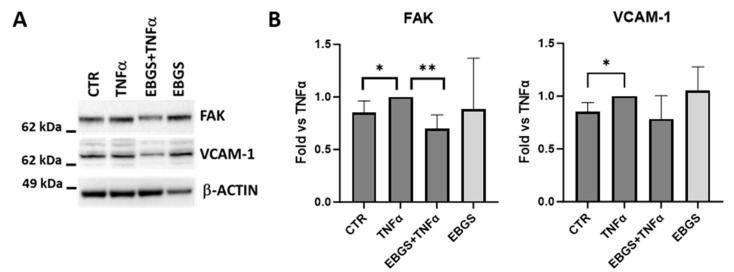
Western blot assay (**A**) and densitometric analysis for FAK and VCAM-1 (**B**) on total extract proteins from HUVEC pretreated or not with EBGS and activated with TNFα. Data are represented as the mean ± SD of three independent experiments. EBGS: escin–bromelain–ginkgo biloba–sage miltiorrhiza. CTR, cells not treated. Statistical analyses were performed using normality test and *t*-test; * *p* < 0.05; ** *p* < 0.005.

In this study, we investigated if the compound EBGS was able to modulate the adhesion pathway, focusing our analysis on vascular cell adhesion molecules 1 (VCAM-1) and focal adhesion kinase (FAK). As we have not found a significant effect of EBGS on the VCAM-1 transcript (Figure 4), we proceeded to evaluate the possible role of the compound in the post-transcriptional modulation. To this aim, we analyzed VCAM-1 and FAK protein levels in HUVEC cells pretreated with EBGS and then stimulated with TNFα. It is interesting to note that the pretreatment of HUVEC cells with EBGS compound inhibits the increase in these proteins induced by TNFα, which is responsible for endothelium activation (Figure 6).

### 2.6. The Presence of Monocyte–Lymphocyte Interaction Strengthens the Adhesion of Platelets to the Endothelial Monolayer, and EBGS Pretreatment Maintains the Ability to Reduce This Interaction

To evaluate if EBGS might exert its activity also in the presence of monocyte activation, we conducted an adhesion assay of platelets, with or without monocyte–lymphocyte population, to the monolayer pretreated for 18 h with the combination of the four compounds (EBGS) and activated with TNFα, as previously described. 

At the end of the incubation, the RNA was extracted and reverse-transcribed into cDNA. Using real-time PCR assays, we evaluated the expression of adhesion molecules involved in the initial platelet interaction to the endothelium, such as P-selectin and platelet membrane glycoprotein GP1b, and the expression of integrins αV and β3, involved subsequently in stronger and stable adhesion of platelets.

**Figure 7:**
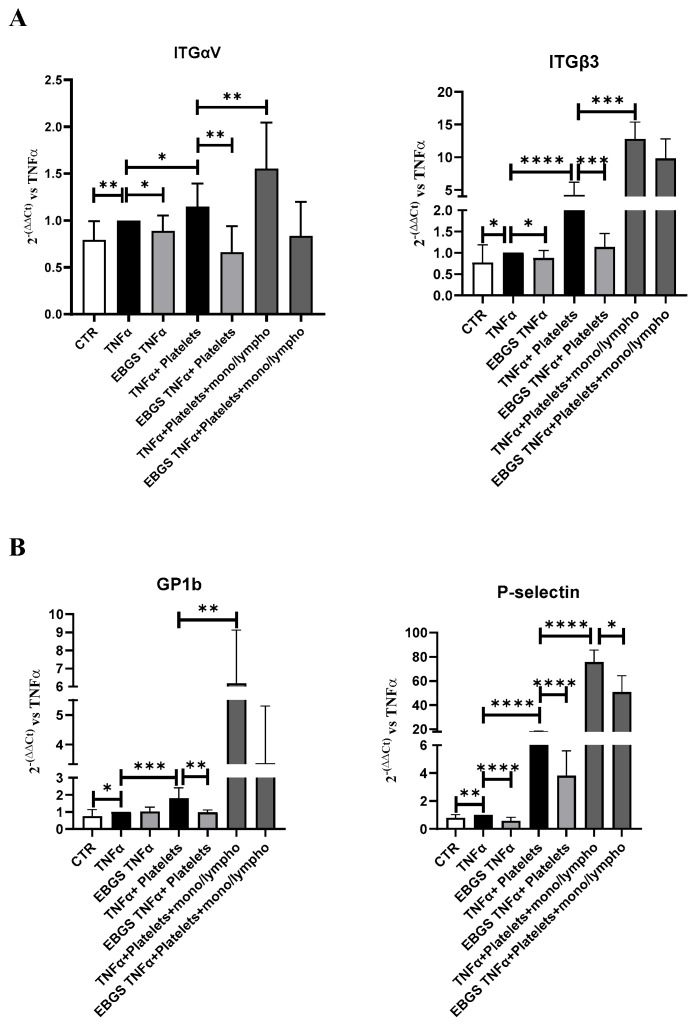
Real-time PCR for integrin αV and β3 (**A**) or platelet membrane glycoprotein GP1b and P-selectin (**B**) in HUVEC pretreated or not with EBGS, activated with TNFα, and finally subjected to platelet adhesion with or without monocyte–lymphocyte population (mono/lympho). Data were normalized for β-actin. ΔΔCt is expressed as a fold of increase (FOI) to the TNFα treated sample. Data are expressed as the mean ± SD of at least three independent experiments. EBGS: escin–bromelain–ginkgo biloba–sage miltiorrhiza. CTR, cells not treated. Statistical analyses were performed using normality test and *t*-test; * *p* < 0.05; ** *p* < 0.005; *** *p* < 0.0005; **** *p* < 0.0001.

The expression of the adhesion molecules analyzed, as shown in Figure 7, increases when the endothelial cells are in the presence of platelets and are reduced in the presence of EBGS, as previously shown. Furthermore, the presence of a monocyte–lymphocyte population significantly increases the expression of these adhesion molecules, confirming that even in our system, the presence of this population stimulates the adhesion of platelets to the endothelial monolayer. Interestingly, the treatment with the EBGS combination significantly reduces the expression of the adhesion molecules evaluated (Figure 7).

## 3. Discussion

Thrombosis is an important factor in diseases such as atherosclerosis, myocardial infarction, stroke, and pulmonary embolism, often causing ischemic damage to tissues and even death [36]. In coronary or cerebrovascular circulation, it is considered the key pathological process underlying acute coronary syndrome and ischaemic stroke, which together represent the leading cause of morbidity and mortality in the world [36,37]. In physiological conditions, endothelial cells in the vessel wall keep platelets at rest by releasing nitric oxide, prostacyclin, ectonucleotidase CD39, and ADPase. However, damaged blood vessels, inflammation, atherosclerosis, and other pathological factors can disrupt this balance [38]. Cardiovascular endothelial cell damage, abnormal blood flow status, and increased blood coagulation have been reported to provide favorable conditions for thrombosis [39,40]. In addition, inflammation in the vascular microenvironment and oxidative stress are also important factors in thrombosis [41,42]. It is known that platelet adhesion, activation, and aggregation represent critical processes in thrombus formation after endothelial damage.

Even if modern medicine has a wide range of anticoagulant drugs, thrombosis still leads to cardiovascular diseases, myocardial infarction, and stroke. Current prevention or treatment drugs that have been used for many years have been replaced by newer ones that show greater potential for the prevention and treatment of thrombosis with modest but gradual improvement. Unfortunately, several drugs used in long-term thrombosis treatment seem to lead to non-desirable side effects, mainly bleeding. For example, some drugs, such as heparin, urokinase, and streptokinase, may increase the risk of bleeding [39,43].

To date, various substances of natural origin are known to reduce the level of oxidative stress and the risk of platelet hyperactivation and may be important in the treatment of thrombotic complications [44,45]. In this paper, we investigated the effects of the combination of four natural compounds (escin, bromelain, ginkgo biloba, sage miltiorrhiza) known to have singularly anti-inflammatory, antioxidant, or anticoagulant effects. We are interested in evaluating their combination (EBGS) to obtain a possible new compound that takes advantage of their combinatory activity. 

Platelet activation is a complex and dynamic process that is mediated by several agonists. When platelets are activated, they promote a conformational change in integrin β3 that mediates an inside–out signaling that activates several proteins through tyrosine phosphorylation, such as FAK (Focal Adhesion Kinase), finally inducing platelet adhesion [46,47]. It is well known that the cytoplasmic tyrosine kinase FAK, thanks to its position at focal adhesions, can interfere with cytoskeleton reorganization in adherent cells and integrin β3 activity [48,49,50]. Moreover, focal adhesion kinases are expressed and activated in blood platelets and could play a crucial role in platelet adhesion and spreading [51,52,53].

It is well known that garcinol, derived from the peel of the fruit Garcinia indica, exerts anticancer effects; moreover, it has an anti-inflammatory and cardioprotective role [54,55]. Cao et al. demonstrated that garcinol can attenuate platelet aggregation by acting on P-selectin abundance and integrin β3 activity [56]. Chih-Wei Hsia and collaborators recently demonstrated that garcinol inhibits fibrinogen–integrin β3 interaction, thus suggesting a potential role of garcinol as a new antithrombotic agent [57]. 

Considering that garcinol affects platelet activation via integrins, we investigate the expression of integrins in our model. Our results demonstrated that the pretreatment of endothelial cell-monolayer with the combination of the four natural compounds, EBGS, reduces the ability of the platelets to adhere to activated endothelium, as proved in the adhesion assay. Moreover, when we pretreated endothelial cells with EBGS, we observed a downregulation of the mRNA level of the molecules involved in the initial step of platelet–endothelium interaction (P-selectin and GP1b) and the stabilization of this interaction (integrins αV and β3). We obtained the same results by carrying out our platelet–endothelium adhesion experiments in the presence or not of the monocyte–lymphocyte cell population. Moreover, we demonstrated that EBGS can affect the protein expression of FAK, whose activity is strictly correlated with that of integrin β3, as discussed above.

The platelet–endothelium interaction and the subsequent adhesion of platelets to the endothelium monolayer are regulated by several adhesion molecules. Vascular cell adhesion protein 1 (VCAM-1) is expressed in endothelial cells in response to cytokines such as TNFα, and it is known to regulate inflammation-associated vascular adhesion [58]. VCAM-1 and ICAM-1 (Intercellular Adhesion Molecule 1) are upregulated in atherosclerosis lesions, but Cybulsky and collaborators demonstrated in mice models that VCAM-1 was involved in the initial stage of atherosclerosis while ICAM-1 was involved in lesion progression [59]. 

For this reason and to better understand the role of our compound EBGS, we decide to analyze the protein expression of VCAM-1. We demonstrated that the pretreatment of endothelial cells with EBGS reduces the protein level of VCAM-1 on endothelial cells after platelet adhesion, demonstrating once again that this combination of natural compounds can modulate different steps of platelet adhesion to endothelium monolayer, potentially affecting the coagulation cascade. 

It is well known that, after an atherosclerotic lesion, activated platelets are recruited to the vascular wall and adhere to the endothelium, inducing the secretion of matrix metalloproteinases (MMPs), such as MMP-2 and MMP-9 [12,13]. Moreover, endothelial cells activate a pro-inflammatory phenotype, inducing the release of inflammatory chemokines such as interleukin 6 (IL6) and interleukin 8 (IL8). 

Huilcaman and coworkers demonstrated that “Endothelial transmigration of platelets depends on soluble factors released by activated endothelial cells and monocytes” [20]. Moreover, Zhang and collaborators demonstrated that Ginkgolide B, a ginkgo biloba leaf extract, inhibits the adhesion of platelets and monocytes to activated endothelium [60]. Here, we demonstrate that the monocyte–lymphocyte population strengthens the adhesion of platelets to the activated endothelium, and EBGS pretreatment maintains the ability to reduce this interaction.

Finally, our data show that the pretreatment of endothelial cells with EBGS reduces the release of inflammatory chemokines and metalloproteinases by activated endothelial cells after platelet interaction. This is an important result, considering the involvement of inflammation as a mechanism resulting in thrombosis.

## 4. Materials and Methods

### 4.1. Preparation of Escin (E), Bromelain (B), Ginkgo Biloba (G), and Sage Miltiorrhiza (S) Extracts and Their Combination EBGS

All four compounds, reduced to powder, were extracted in ethanol 65–75% *v*/*v* by Nuova Farmaceutica (Carruba di Riposto, CT, Italy), which also made several chemical analyses and quality controls to check for bacterial or yeast and mold contamination.

Escin (E) is a mixture of acylated triterpene glycosides isolated from the seeds of horse chestnut (*Aesculus hippocastanum* L.). Its appearance is almost white and amorphous powder. The compound used for our experiments is composed of β-escin and α-escin in a ratio of 2:1. The powder was solubilized in a solution of 50% DMSO in PBS to a final 25 mg/mL concentration. 

Bromelain (B) is the major sulfhydryl proteolytic enzyme found in pineapple plants (*Ananas comosus*). Its appearance is off-white to cream, hygroscopic powder. It was solubilized in a solution of 50% DMSO in PBS to a final 50 mg/mL concentration.

Ginkgo biloba (G) was extracted from the leaves of *Ginkgo biloba* L. Its appearance is a brown and fine powder. It was solubilized in a solution with 0.5% DMSO in an experimental cell medium (1 volume of vascular cell basal medium and 9 volumes of RPMI 1640) up to a final concentration of 80 μg/mL.

Sage miltiorrhiza (S) was extracted from the root of sage miltiorrhiza. Its appearance is a dark and fine powder. It was solubilized in an experimental cell medium (9 volumes of vascular cell basal medium and 1 volume of RPMI 1640) up to a final concentration of 400 μg/mL.

The combination EBGS is the mix of each 4 extracts in equal amounts (0.25 μg/mL of E, 0.25 μg/mL of B, 0.25 μg/mL of G, 0.25 μg/mL of S).

### 4.2. Cell Culture and Treatment Procedure

HUVEC (Human Umbilical Vein Endothelial cells) were obtained from ATCC and grown in vascular cell basal medium supplemented with the Endothelial Cell Growth Kit (ATCC, LGC standards, MI, Italy). The cell line was maintained in a humidified 5% CO2 atmosphere at 37°C. For all the experiments, cells were used at an early passage, following the good practice outlined in the Guidelines for the Use of Cell Lines in Cancer Research. Moreover, cell lines used are certified as being the designated type and have been checked to ensure they are free of contamination. According to the results of the MTT assay (described below), for all experiments, HUVEC were treated as follows: cells were seeded to reach 60% of confluence on the day of the treatment. Cells were treated overnight (o.n.-18 h) with the single compound or with their combination (EBGS, 0.25 μg/mL of each of the 4 compounds) in an experimental cell medium (1 volume of vascular cell basal medium and 9 volumes of RPMI 1640, Euroclone, UK), and finally exposed or not to TNFα (20 ng/mL) for 2 h. The solvent of each compound (DMSO or experimental cell medium) was used as a control.

### 4.3. Viability Assay (MTT Assay)

HUVECs were seeded and treated, as mentioned. Briefly, cells were seeded in a 24-well plate and, the day after, treated or not for 24–48 h with increasing concentration of each compound: 1–5–10–25–50 μg/mL of escin or bromelain; 1–5–10–25 μg/mL of ginkgo biloba or sage miltiorrhiza. In different combinations, HUVEC cells were treated with 0.25 μg/mL of each compound, as described above. At the end of the treatment, the methyl-thiazol-tetrazolium (MTT) assay was performed according to the manufacturer’s instructions (Invitrogen, Thermo Fisher Scientific). Means and standard deviations generated from three independent experiments are reported as the percentage of growth versus control.

### 4.4. RNA Isolation and Real-Time PCR

HUVECs were seeded and treated as mentioned. RNA was isolated using the Illustra RNA spin Mini Isolation Kit (GE Healthcare; Chicago, IL, USA) according to the manufacturer’s instructions. Total RNA from HUVEC was reverse-transcribed to cDNA using the High-Capacity cDNA Reverse Transcription kit (Applied Biosystems, Foster City, CA, USA). For quantitative SYBR Green Real-time PCR, the reaction was carried out in a total volume of 20 µL containing 2X SYBR Green I Master Mix (Applied Biosystems), 2 µL of cDNA, and 100 nM forward and reverse primers. The oligonucleotides used are reported in Table 1.

Real-time PCR was performed in 48-well plates using the Step-One Real-Time PCR System (Applied Biosystems). Relative changes in gene expression between control and treated samples were determined using the ∆∆Ct method. Levels of the target transcript were normalized to a β-Actin endogenous control, constantly expressed in all samples (∆Ct). Additional subtractions were performed between treated samples and control ∆Ct values for Ct values. Final values were expressed as fold change.

### 4.5. Human Platelet Purification

Platelets were isolated from the peripheral blood of healthy patients or patients undergoing hematological controls but not undergoing therapy. Informed consent was obtained from patients (n = 10), according to the Declaration of Helsinki, and with hospital Ethics Committee approval (report n° 01/2023, approved 25 January 2023). The blood sample was centrifuged 10′ at 800 rpm; the isolated plasma obtained was subsequently centrifuged for 3′ at 2000 rpm, and the pellet was resuspended in the experimental cell medium for the subsequent experiments.

### 4.6. Adhesion Assay of Human Platelets on HUVEC Monolayer

HUVECs were seeded and treated as mentioned. At the end of the incubation with TNFα, platelets were added to the activated monolayer at a concentration of 3 × 10^7^/well and left to adhere in the incubator (37 °C and 5% CO_2_) for 1 h. Adherent cells were stained with hematoxylin/eosin. Each test group was assayed in triplicate; six fields for each condition were photographed by phase-contrast microscopy and counted using ImageJ software. The results were expressed as the number of platelets adhered to endothelial cells/number of total platelets for each field.

### 4.7. Enzyme-Linked ImmunoSorbent Assay (ELISA) Assays

HUVEC were seeded at 3.5 × 10^5^ cells per well in 6-well plates and treated as described above. At the end of the experimental time, the conditioned medium was collected and centrifuged to remove cellular debris. The ELISA assays were then performed according to the manufacturer’s instructions. 

The amounts of VEGF, IL6, IL8, MMP-2, and MMP-9 in culture supernatants were determined by using a human-specific ELISA kit (cat. number KHG0111, cat. number KHC0061, cat. number KHC0081, cat. number KHC3081, cat. number BMS2016–2, Thermo Fisher Scientific, Cambridge, MA, USA) and expressed as pg/mL of protein secreted in the cell medium.

### 4.8. Western Blot Analysis

SDS-PAGE and Western blotting were performed according to standard protocols. Briefly, cells were washed in PBS and lysed for 1 h in lysis buffer containing 15 mM Tris/HCl pH 7.5, 120 mM NaCl, 25 mM KCl, 1 mM EDTA, 0.5% Triton X100, and protease inhibitor cocktail (100X, Sigma-Aldrich, St. Louis, MO, USA). Cell debris was removed by centrifugation at 17,000 rpm for 20 min at 4 °C. The protein concentrations were determined by the Bradford microassay method (Sigma-Aldrich) using bovine serum albumin (BSA, Sigma-Aldrich) as a standard. A total of 20 μg protein from each sample was separated using Bolt Bis-Tris gel 4–12% (Thermo Fisher Scientific) and transferred on nitrocellulose membranes (GE Healthcare). The membrane was then blocked in 5% BSA solution (5% BSA, 20 mM Tris, 140 mM NaCl, 0.1% Tween-20) and probed overnight with specific primary antibodies: anti-β-Actin (1:1000, cat. number sc-398103) and anti-FAK (1:500, cat. number sc-1688) from Santa Cruz Biotechnology (Dallas, TX, USA); anti-VCAM-1 (1:500, cat. number PA5–86042), from Thermo Fisher Scientific. The membranes were incubated with mouse or rabbit HRP-conjugated secondary antibody (1:5000, Thermo Fisher Scientific), and the signal was detected by the Chemidoc Biorad acquisition instrument. The obtained images were analyzed using the Image Lab software (Bio-Rad; Hercules, CA, USA).

### 4.9. Human Monocyte–Lymphocyte Fraction Purification

The monocyte–lymphocyte population was isolated from the peripheral blood of patients (n = 10). Informed consent was obtained from patients, according to the Declaration of Helsinki, and with hospital Ethics Committee approval. The blood sample was diluted in 1 volume of phosphate buffer (PBS, Euroclone, UK), stratified with 1.5 volumes of Ficoll-paque (Merk Life Science S.r.l., Milan, Italy), and centrifuged 30′ at 400× *g*. The ring recovered, containing monocytes and a small percentage of lymphocytes, was washed in PBS two times and resuspended in RPMI medium.

### 4.10. Adhesion Assay of Human Platelets and Human Monocyte–Lymphocyte on HUVEC Monolayer

HUVECs were seeded and treated as mentioned. At the end of the incubation with TNFα, platelets were added to the pretreated and activated monolayer at a concentration of 3 × 10^7^/well with or without monocyte–lymphocyte population at the concentration of 3 × 10^5^/mL and left to adhere in the incubator (37 °C and 5% CO_2_) for 1 h. The platelets: monocytes ratio of 100:1 was set according to previous publications [20,61]. At the end of incubation, RNA was isolated and reverse-transcribed to cDNA, as described in “4.4 RNA Isolation and Real-Time PCR”.

### 4.11. Statistical Analysis

Data are presented as mean ± standard deviation (SD) of independent biological replicates *n* ≥ 3. The normal data distribution was assessed by the Shapiro–Wilk test. The data obtained in all the experiments followed a normal distribution; thus, the statistical significance of differences was analyzed using a one-sample *t*-test to compare the mean to a hypothetical mean or a two-tailed Student’s *t*-test to compare the group. A *p*-value ≤ 0.05 was considered significant. Statistical analyses were performed using GraphPad Prism 10 software (GraphPad Software, San Diego, CA, USA). *p*-values were indicated in the graphs.

## 5. Conclusions and Limitations

To date, high hopes are associated with compounds of natural origin, which show anticoagulant effects both at the experimental and clinical stages and can be used in the prophylactic or supportive methods of treatment of thrombotic diseases. 

The use of natural compounds as an alternative or supporting therapies is very attentive, especially in fields such as the one covered by our study. This is because anticoagulant therapies often have significant adverse effects in many patients. Although the combination we proposed, EBGS, showed encouraging results, it is clear that many other studies still need to be conducted to evaluate the possible use of EBGS as a supportive therapy to conventional ones. 

Another possible limitation is due to the small number of patients recruited for our study. For our experiments, we used human samples and, in particular, the peripheral blood of healthy patients or patients undergoing hematological controls but not undergoing therapy. The difficulty was linked to the fact of recruiting patients who went through hematological controls without having any anticoagulant therapy. It is well known that the larger the sample, the more precise your results will be. However, even if the number of samples is not very high, we obtained statistically significant results. 

Moreover, we decided to take a few ml from patients to avoid being too invasive. This determined that we obtained few materials and could not perform many other experiments.

Data presented in this paper suggest the possible use as supportive therapy of the new drug formulation EBGS, derived from the combination of the four natural compounds, escin, bromelain, ginkgo biloba, and sage miltiorrhiza. These compounds singularly had no significant effects on the modulation of the platelet–endothelium interaction, but their combined action reduced platelet adhesion to activated endothelium, thus possibly preventing coagulation cascade initiation.

## 6. Patents

There are no patents resulting from the work reported in this manuscript.

## Figures and Tables

**Table 1:** The primers’ sequences used for gene expression analysis.

Primers	Forward (5′-3′)	Reverse (5′-3′)
p-selectin	TCCGCTGCATTGACTCTGGACA	CTGAACGCTCTCAAGGATGGAG
integrins β3	CATGGATTCCAGCAATGTCCTCC	TTGAGGCAGGTGGATTGAAGG
integrins αV	AGGAGAAGGTGCCTACGAACT	GCACAGGAAAGTCTTGCTAAGGC
GP1b	ACCATCCTGGTGTCTGCCACAA	ACGGAGCTTTGGTGGCTGATCA
β-actin	CAAGAGATGGCCACGGCTGCT	TCCTTCTGCATCCTGTCGGCA

## Data Availability

Data will be made available on request.

## Abbreviations

HUVEC: human vein endothelial cells; EBGS: escin, bromelain, ginkgo biloba, sage miltiorrhiza; TNFα: tumor necrosis factor α; MMP-2: metalloproteinase 2; MMP-9: metalloproteinase 9; ECs: endothelial cells; E: escin; B: bromelain; G: ginkgo biloba; S: sage miltiorrhiza; GP1b: platelet membrane glycoprotein 1b; IL-6: Interleukin 6; IL-8: Interleukin 8; VCAM-1: vascular cell adhesion molecules 1; ICAM-1: Intercellular Adhesion Molecule 1; FAK: focal adhesion kinase; ITGαV: Integrin αV; ITGβ3: Integrin β3.
